# Assessment of Psychosocial Functioning of Polish Nurses during COVID-19 Pandemic

**DOI:** 10.3390/ijerph19031435

**Published:** 2022-01-27

**Authors:** Kamila Rachubińska, Anna Maria Cybulska, Joanna Sołek-Pastuszka, Mariusz Panczyk, Marzanna Stanisławska, Przemysław Ustianowski, Elżbieta Grochans

**Affiliations:** 1 Department of Nursing, Pomeranian Medical University in Szczecin, 71-210 Szczecin, Poland; kamila.rachubinska@pum.edu.pl (K.R.); stamarz@pum.edu.pl (M.S.); przemyslaw.ustianowski@gmail.com (P.U.); elzbieta.grochans@pum.edu.pl (E.G.); 2 Department of Anaesthesiology and Intensive Therapy in Szczecin, Pomeranian Medical University, 71-242 Szczecin, Poland; joanna.pastuszka@pum.edu.pl; 3 Department of Education and Research in Health Sciences, Faculty of Health Science, Medical University of Warsaw, 00-581 Warsaw, Poland; mariusz.panczyk@wum.edu.pl

**Keywords:** public health, COVID-19, pandemic, depression, anxiety, insomnia, nursing

## Abstract

(1) The COVID-19 pandemic has significantly affected the psychological well-being of people around the world. The aim of this study was to assess the levels of psychological distress of nurses (anxiety, depression, stress, insomnia) in relation to sociodemographic variables and psychosocial variables: self-assessment of health, quarantine, psychological support, presence of chronic diseases and the Impact of Events Scale (IES-R). (2) A total of 207 nurses working with COVID-19 patients at the Independent Public Clinical Hospital No. 1 of the Pomeranian Medical University in Szczecin participated in the study. The study was conducted with the diagnostic survey method, using the Athens Insomnia Scale, the Generalized Anxiety Disorder questionnaire, the Impact of Event Scale—Revised, the Patient Health Questionnaire-9, The Perceived Stress Scale and a questionnaire of our authorship. (3) Among the respondents, 40.58% suffered sleep disturbance, 36.71% had mild anxiety, 71.95% had high stress according to the PSS-10 and 31.88% had depression according to the PHQ-9. The study observed that the chances of insomnia decreased with the age of the respondents. Moreover, the form of employment of nurses significantly affected the levels of depression, anxiety and stress. (4) Education, gender and age were variables that significantly affected the severity of anxiety, depression and insomnia in the surveyed nurses working with patients with COVID-19.

## 1. Introduction

The COVID-19 pandemic has affected the well-being of people around the world, and from the beginning of the pandemic, specialized bodies have emphasized the importance of protecting the health of those that are particularly vulnerable to SARS-CoV-2 infection, including medical workers. The changes that occurred as a result were multifaceted and affected not only society as a whole but, more importantly, each individual. In a very short period, almost every person had to reorganize their daily functioning, and the chaos in the public space translated into the loss of daily routine activities. The very fact of being threatened by the danger of infection with an unknown and dangerous pathogen and the complete change in daily functioning undoubtedly became a source of stress. The fear of losing one’s own and one’s family’s health and life, the vision of potential life and economic problems and the periods of forced isolation negatively influenced the well-being of most of us. It seems that the stress associated with new conditions of functioning may have significantly affected people for whom everyday professional activity was a potential source of infection with an unknown virus. Working in healthcare during this specific period became particularly difficult. The lack of reliable information and detailed procedures, limitations related to the scarcity of protective measures and, perhaps most importantly, initial diagnostic difficulties resulting from the lack of access to virus-confirming tests and long waiting periods for their results caused tension, fatigue and a sense of overload. These elements may have resulted in symptoms of anxiety, depression, sadness and insomnia [1,2].

The definition of anxiety is not unequivocal in the literature, although the descriptive characteristics are well circumscribed and easily identifiable. The American Psychiatric Association defines anxiety as the anticipation of future danger or a negative event, accompanied by feelings of dysphoria or physical symptoms of tension [3]. The term “depression” is used to describe a particular type of mood and emotion disorder, recognized as a disease phenomenon. In fact, there is no fully sharp line between “ordinary” depression and “true” depression. Depression is characterized by the presence of sadness and despair for a long period that affects the disorganization of complex activity [4]. Stress is the process by which environmental factors threaten or disturb the body’s balance and by which the body responds to the threat. The environmental factors in question are often referred to as stressors. They activate complex mechanisms of both physiological and psychological responses and significantly affect the health status of an individual [5].

Presumably, exposure to deep, prolonged stress during the COVID-19 pandemic will have a negative impact on the mental health of the public, including healthcare workers. Workers in direct contact with COVID-19 patients are particularly vulnerable to symptoms such as depression, anxiety, stress and poor sleep quality, and their mental health may require special attention. In the fight against the COVID-19 pandemic, medical workers often have to live with the risk of infection, often inadequately protected, overworked, exhausted, sleep-deprived, isolated and lacking contact with loved ones. The difficult situation often has a negative impact on their levels of psychological distress, which may result in a decrease in the quality of their work. In the face of these threats, it is necessary to implement a comprehensive mental health plan, especially among workers who have direct contact with the infected [6]. The aim of the study was to assess the levels of psychological distress of nursing staff (anxiety, depression, stress, insomnia) in relation to sociodemographic and psychosocial variables: self-rated health status, quarantine, psychological support, presence of chronic diseases and experience of traumatic events according to the Impact of Events Scale (IES-R) during the COVID-19 pandemic.

## 2. Materials and Methods

### 2.1. Settings and Design

A diagnostic survey method utilizing a questionnaire technique was used to assess the levels of psychological distress of nurses. Due to the COVID-19 pandemic and the recommendations of the Polish government to minimize contact with other people, potential respondents were invited via email to participate in the study. Volunteers completed the survey questionnaires in Polish via an online platform (https://docs.google.com/ (accessed on 20 November 2021)) (Table S1).

Respondents were recruited from among nursing staff working directly with patients diagnosed with SARS-CoV-2 infection in the Independent Public Clinical Hospital No. 1 of the Pomeranian Medical University named Prof. Tadeusz Sokołowski in Szczecin.

The inclusion criteria for the study were a current license to practice as a nurse, aged >18 years and informed consent to participate in the study. The study was conducted according to the guidelines of the Declaration of Helsinki and approved by the Ethics Committee of the Pomeranian Medical University in Szczecin (KB-0012/25/04/2020/Z). Our study was conducted taking into account ethical considerations. Informed consent was required, and participation in the study was voluntary. Moreover, the participants were assured of anonymity and confidentiality, and were free to withdraw from the study at any stage.

The results of the study were presented to the Chief Nurse of SPSK 1 in Szczecin. Due to the anonymity of the study participants, we requested a more complete analysis of psychological disorders and psychological nurses for psychological support. 

### 2.2. Research Instruments

Standardized survey instruments were used to assess the psychosocial functioning of nursing staff during the COVID-19 pandemic:

- **The Impact of Event Scale (IES-R, Impact of Event Scale—Revised)** [7] is a questionnaire designed to determine the extent of psychological impact following exposure to a public health crisis within one week of exposure. The Impact of Event Scale consists of 22 statements describing symptoms of perceived stress in the last 7 days in relation to the traumatic event experienced. It is rated on a 5-point Likert-type scale (0–4). It captures three dimensions of PTSD: intrusion (expressing recurrent images, dreams, thoughts or perceptual impressions associated with the trauma), arousal (characterized by increased alertness, anxiety, impatience and difficulty concentrating) and avoidance (manifested by efforts to rid oneself of thoughts, emotions or conversations associated with the trauma). Cronbach’s alpha coefficient for the entire scale, which finally included 12 items, was 0.77. A three-factor structure of the tool was demonstrated, explaining 60.04% of the variance. This analysis revealed moderate-to-high values of the factor loadings of all items that form subscales, with the exception of the fifth subject. On this basis, it was decided to reject the fifth item. The Polish version of the PDI is a relevant and reliable distress assessment tool.
- **The Generalized Anxiety Questionnaire** (GAD-7, Generalized Anxiety Disorder) is a screening tool used to determine feelings associated with generalized anxiety syndrome. The survey consists of 7 questions. Each question has a score from 0 to 3 points, the sum of which indicates the severity of anxiety: 0–4 points (no anxiety), 5–9 (mild anxiety), 10–14 (moderate anxiety) and 15–21 (severe anxiety). Using the threshold score of 10, the GAD-7 has a sensitivity of 89% and a specificity of 82% for generalized anxiety disorder. It is also moderately good at screening for other anxiety disorders: panic disorder (sensitivity 74%, specificity 81%), social anxiety disorder (sensitivity 72%, specificity 80%) and post-traumatic stress disorder (sensitivity 66%, specificity 81%) [8].
- **The PHQ-9** (Patient Health Questionnaire-9) is a questionnaire that was designed to screen for depression. It was developed based on the diagnostic criteria for depression contained in the Diagnostic and Statistical Manual of Mental Disorders (DSM-IV). The maximum number of points possible is 27, which indicates the highest possible severity of depression. A score of less than five indicates normal, 5 to 9 points indicates mild depression, 10 to 14 points indicates moderate depression, 15 to 19 points indicates moderately severe depression and 20 to 27 points indicates severe depression. The PHQ-9 showed significant positive internal consistency (Cronbach’s alpha = 0.7) and the scores of each of its nine items positively correlated (0.31–0.68; *p* < 0.05) with the total score. The convergent validity was significantly positive (r = 0.58; *p* < 0.05). Using >6 points as the cutoff point, the sensitivity and specificity of the PHQ-9 for recognizing a major depression episode were found to be 70.4% and 78.2% respectively [9].
- **The Athens Insomnia Scale** (AIS) is a short, eight-item scale that allows for quantitative measurement of insomnia symptoms based on ICD-10 criteria. The total score of the scale ranges from 0 to 24 points. The first five items relate to sleep-related symptoms (difficulty falling asleep, waking up during the night, waking up early in the morning, sleep duration and quality) and correspond to criterion A of the ICD-10 diagnosis of inorganic insomnia. A symptom should be marked if it occurred at least three times a week for at least a month, which is consistent with the duration and frequency of symptoms required for the ICD-10 diagnosis of insomnia (criterion B). The other three items relate to daytime functioning (mood, physical and mental performance, sleepiness) and correspond to criterion C of the ICD-10 diagnosis of insomnia, which includes complaints about the consequences of insomnia during the day. The Athens Insomnia Scale is the first tool for assessing insomnia-related symptoms, which has achieved Polish validation. The study confirmed the good psychometric properties of the scale. The internal consistency (Cronbach’s alpha = 0.90) and the test–retest reliability (r^2^ = 0.92) of the AIS were found to be very satisfactory. These values remained practically unchanged when any of the items were removed from the analysis [10].
- **The Perceived Stress Scale PSS-10** (PSS-10, The Perceived Stress Scale) is an instrument that is used to assess the severity of stress related to the subject’s situation in the last 4 weeks in the context of subjective feelings and personal life problems. Interpretation is carried out by analyzing the ten-point norms that indicate the severity of stress: low score (1–4 points), average score (5–6 points) and high score (7–10 points). The Polish adaptation of the scale was made in 2009 by Juczyński and Ogińska-Bulik. The reliability of the Polish adaptation of the PSS-10 scale (Cronbach’s α coefficient) ranges from 0.72 to 0.90 [11].
- **Self-administered questionnaire—including questions about sociodemographic data** (age, education, place of residence, marital status, employment status, parental status), physical symptoms over the past 14 days (included fever, chills, headache, muscle aches, cough, difficulty breathing, dizziness, sore throat and persistent fever, as well as persistent fever and cough or difficulty breathing), history of contact with COVID-19 (close contact with a person with confirmed COVID-19).

### 2.3. Statistical Analysis

Quantitative and categorical variables were described with descriptive statistics methods. For the quantitative variables, the following measures were determined: central tendency (mean, M) and dispersion (standard deviation, SD). For the categorical variables, the following measures were determined: number (N) and frequency (%).

The influences of selected sociodemographic and health-related factors and the psychological impact after exposure on insomnia according to the IES-R were estimated using a non-linear estimation for the logistic regression model. The Rosenbrock and quasi-Newton methods of estimation were applied, appointing asymptotic standard errors. The accuracy of the data adjustment to the suggested logit was checked using the Hosmer–Lemeshow test. The model fit was also tested using V-fold cross-validation. For each factor, the odds ratio (OR) was determined, together with a 95% confidence interval.

The influence of selected sociodemographic and health-related factors, as well as the psychological impact after exposure according to IES-R, on the levels of psychological distress of nurses (anxiety, depression, stress) was estimated using a multivariate linear regression model. The least-squares method of estimation was applied to calculate the parameters of the regression model. For each factor, which was an independent variable tested in the regression model, the following indicators were determined: unstandardized (b) and standardized regression (β_stand._) coefficients, 95% confidence interval (CI), t-test and *p*-value. The proportion of the variance for a dependent variable that was explained by the independent variables was calculated with the adjusted R-squared (R^2^_adj._).

All the calculations were performed with STATISTICATM 13.3 software (TIBCO Software, Palo Alto, CA, USA). For all analyses, a *p*-level of <0.05 was considered statistically significant.

## 3. Results

### 3.1. Characteristics of Respondents

A total of 312 respondents working directly with patients diagnosed with COVID-19 were invited to participate in the survey between 1 January and 1 April 2021. Only 207 nurses correctly completed the surveys (completion rate: 66%). The mean age of the respondents was 37.87 years. The vast majority of the respondents were female (83.09%), in a formal relationship (52.66%), achieved higher education (79.23%) and living in a city of more than 100 thousand residents (66.67%). The most frequently chosen forms of employment were employment contract (67.15%) and non-shift work (68.60%). Half of the respondents had children (50.24%). A majority of the respondents did not have any chronic disease (68.60%), 12.56% had chronic hyperthyroidism or hypothyroidism and 7.25% had hypertension (Table S2).

### 3.2. Pandemic-Related Variables

Most of the respondents (65.70%) rated their health as good and had not been quarantined (80.68%). Almost all (98.55%) respondents stated the presence of personal protective equipment, while more than half of the respondents (57.49%) indicated a lack of psychological support in the workplace. Less than half of the respondents (48.79%) indicated close contact with a person with confirmed COVID-19 infection in the past 4 weeks. The most common symptom (40.10%) in the past 4 weeks was a headache.

### 3.3. Psychological Variables

Selected psychological variables (sleep disturbance, anxiety, stress severity, depression) of the respondents were analyzed in this study.

Based on the results obtained according to the Athens Insomnia Scale (AIS), it was found that 40.58% of the respondents had sleep disorders. When anxiety was examined according to the Generalized Anxiety Questionnaire (GAD-7), it was observed that the vast majority of the respondents experienced anxiety of varying severities: 36.71% had mild anxiety, 19.81% had moderate anxiety and 9.18% had severe anxiety. Moreover, it was shown that a majority of the respondents (71.95%) experienced a high level of stress according to PSS-10, while in the case of depression, according to the PHQ-9, it was shown that only 37.2% of the respondents had no symptoms.

The psychological impact of the COVID-19 pandemic was assessed using the results obtained according to the IES-R. It was observed that the mean total score was 34.25 points (SD = 19.65). For the subscales, the respondents scored 12.34 points, 11.58 points and 12.12 points for intrusion, arousal and avoidance, respectively (Tables S3 and S4).

### 3.4. Assessment of Nurses’ Levels of Psychological Distress (Anxiety, Depression, Stress, Insomnia) in Relation to Sociodemographic Variables

This study analyzed the influence of selected sociodemographic variables (age, gender, marital status, place of residence, education, form of employment) on the psychological variables of nurses during the SARS-CoV-2 pandemic.

Based on the collected data, it was observed that the chance of experiencing insomnia decreased with the age of the subjects (OR = 0.959, *p* = 0.023) (Table 1).

Model I explained approximately 9% of the variance in the Generalized Anxiety Disorder (GAD-7) anxiety variable (F(13, 193) = 2.614, *p* = 0.002). Respondents with a college education had significantly higher levels of anxiety according to GAD-7 compared with those with secondary/post-secondary education (β_stand._ = 0.190, *p* = 0.014). Respondents employed under a contract of employment had significantly higher levels of anxiety as measured by the GAD-7 compared with people without such a form of employment (β_stand._ = 0.400, *p* = 0.002), while those employed under a contract of employment also had significantly higher levels of anxiety as measured by the GAD-7 compared with persons without such a form of employment (β_stand._ = 0.328, *p* = 0.015). Respondents employed on the basis of a contract of mandate showed a significantly higher level of anxiety according to GAD-7 in comparison with persons without this form of employment (β_stand._ = 0.169, *p* = 0.037) (Table S5).

In the case of model II, which explained more than 7% of the variation in depression using the Patient Health Questionnaire-9 (PHQ-9) (F(13, 193) = 2.105, *p* = 0.015), men had a significantly lower level of depression compared with women (β_stand._ = −0.154, *p* = 0.030). Respondents employed under a contract of employment had significantly higher levels of depression according to the PHQ-9 compared with those without this form of employment (β_stand._ = 0.415, *p* = 0.002). Respondents employed under a contract had significantly higher levels of depression compared with those without this form of employment (β_stand._ = 0.299, *p* = 0.029), while those employed under a contract of mandate had significantly higher levels of depression compared with those without this form of employment (β_stand._ = 0.218, *p* = 0.008) (Table S6).

Model III explained only 0.2% of the variation in the stress level variable according to The Perceived Stress Scale (PSS-10) (F(13, 193) = 1.309, *p* = 0.050). Respondents employed under a contract of employment had significantly higher levels of stress compared with those without this form of employment (β_stand._ = 0.347, *p* = 0.011) (Table S7).

### 3.5. Assessment of Levels of Psychological Distress of Nurses (Anxiety, Depression, Stress, Insomnia) in Relation to Self-Assessment of Health, Quarantine, Psychological Support, Presence of Chronic Diseases and Psychological Impact after Exposure according to the IES-R

This study analyzed the effects of selected COVID-19 pandemic-related variables (self-rated health status, having served in quarantine, psychological support, presence of chronic illnesses and post-exposure psychological impact according to IES-R) on nurses’ psychological variables during the SARS-CoV-2 pandemic.

Based on the collected data, it was observed that the chance of insomnia increased with the increase in the severity of the trait “agitation” according to the IES-R (OR = 1.217, *p* = 0.002) (Table 2).

Model IV explained approximately 40% of the variance in the anxiety variable according to the Generalized Anxiety Disorder (GAD-7) questionnaire (F(8, 198) = 18.387, *p* < 0.001). Those in quarantine had significantly higher levels of anxiety than those who did not have this experience (β_stand._ = 0.123, *p* = 0.026). Study nurses who rated their health as very good had significantly lower levels of anxiety than those who rated their health as poor or average (β_stand._ = −0.132, *p* = 0.023). As the level of intrusiveness according to the IES-R of the subjects increased, the level of anxiety according to GAD-7 increased significantly (β_stand._ = 0.340, *p* = 0.001); furthermore, the increase in the level of agitation according to the IES-R caused a significant increase in the level of anxiety (β_stand._ = 0.366, *p* = 0.002) (Table 3).

Model V explained approximately 41% of the variation in the variable of depression according to the Patient Health Questionnaire-9 (PHQ-9) (F(8, 198) = 22.715, *p* < 0.001). Study nurses who rated their health as very good had significantly lower levels of depression according to the PHQ-9 than those who rated their health as poor or average (β_stand._ = −0.147, *p* = 0.011). Among the respondents, as the level of intrusiveness according to the IES-R increased, the level of depressiveness measured using the PHQ-9 increased significantly (β_stand._ = 0.387, *p* < 0.001); furthermore, the increase in the level of arousal according to the IES-R caused a significant increase in depressiveness (β_stand._ = 0.347, *p* = 0.004) (Table 4).

Model VI explained approximately 24% of the variance in the stress variable according to The Perceived Stress Scale (PSS-10) (F(8, 198) = 9.287, *p* < 0.001). Individuals who rated their health as good had significantly lower levels of stress than those who rated their health as poor or average (β_stand._ = −0.149, *p* = 0.017). As the intrusion level according to the IES-R of the subjects increased, the stress level according to the PSS-10 increased significantly (β_stand._ = 0.281, *p* = 0.016) (Table 5).

## 4. Discussion

The years 2020 and 2021 in Poland became a challenging period due to the startling changes in social functioning that occurred with the announcement of the SARS-CoV-2 pandemic. As many as 8000 to 12,000 cases of coronavirus infection were reported daily between 1 and 4 January 2021. More than half of the beds designated for COVID-19 patients were occupied. During this period, a significant number of people struggled with symptoms of tension, anxiety, restlessness and sleep disturbances [12,13]. Healthcare workers were particularly vulnerable to stress symptoms. According to scientific analyses, mood and sleep disorders were most common in this occupational group during the pandemic period [14,15].

According to the results of our study, most of the nurses surveyed scored highly on the perceived stress scale. More than half of the respondents demonstrated moderate and mild levels of anxiety and almost 10% demonstrated severe anxiety symptoms. Almost half of the nurses surveyed suffered from sleep disorders. Almost one-third of the respondents showed full depressive symptoms and another one-third of the respondents showed mild depressive symptoms. An online survey of a group of physicians conducted in Turkey in March 2020 showed that more than half of the medics experienced symptoms of anxiety and depression [16]. Similar results were obtained by other researchers that examined the severity of stress of people working in hospitals during the COVID-19 pandemic [17,18,19,20,21]. Similar results were obtained by Nakhostin; however, in their scientific report, the analysis included a group of medical students [22].

According to the results of our study, more than half of the respondents indicated a lack of psychological support in the workplace. According to the study of Kang et al. [1], in terms of psychological support, 36.3% of the respondents received written psychoeducational materials (i.e., pamphlets, brochures and books), half of the respondents received psychological support through the media (which included online psychological support, as well as information obtained through television and online platforms) and 17.5% participated in group psychological counseling. It was noted that the higher the level of exposure to COVID-19 patients, the more severe the mental health disorders [23].

The nursing professionals of a COVID-19 team have significant levels of anxiety, depression and stress, and the factors associated with depression and stress were identified [24]. According to the study by Appel et al., the nursing professionals of the COVID-19 team studied had significant levels of anxiety, depression and stress, and the factors associated with depression and stress were identified [25].

According to a meta-analysis by Al Maqubali that estimated the combined prevalence of stress, anxiety, depression and sleep disturbance among nurses during the COVID-19 pandemic, the combined prevalences of stress, anxiety, depression and sleep disturbance (43%, 37%, 35% and 43%, respectively) among nurses during the COVID-19 outbreak suggested that at least one-third of nurses experienced stress, anxiety, depression and sleep disturbance. These findings are higher than those reported in the general population during the same period [26]. Shi et al. reported that, in the general population, 24% had stress, 32% had anxiety, 28% had depression and 29% had insomnia. This was because nurses were more likely to be patients with COVID-19 [27].

Lai et al. [28] conducted a cross-sectional, questionnaire-based study of the mental health of workers exposed to direct contact with COVID-19 patients. The results indicated that depressive and anxiety symptoms were present in half of the respondents, insomnia symptoms were present in over one-third of the respondents and distress as measured by the IES-R was present in nearly three-quarters of the respondents. Higher rates of anxiety symptoms were reported in women compared with men. Frontline workers, i.e., those involved in the direct diagnosis, treatment and care of patients with COVID-19, had a higher risk of depressive symptoms, insomnia and distress. The above results are consistent with those of Ten et al. [29] and Chew et al. [30].

The research work of Yin and Zeng [31] focused on the needs of nursing staff members. Qualitative analyses revealed the predominant needs for maintaining health and safety, needs for interpersonal relationships and warmth, concern from the community and needs for knowledge about COVID-19.

In Li’s study [32], the most significant finding was that nursing staff who did not work directly with patients with COVID-19 had higher levels of vicarious traumatization compared with those who worked on the front line of medicine. According to Li et al. [33], this may have been due to their psychological preparation, the work experience of this group and they volunteered to work on the front line. The authors recommend that those medical workers who are not directly involved in the treatment of patients with COVID-19 should also be included in the support offered.

Our results indicated that men had significantly lower levels of depression compared with women. Similar results were obtained by Albert et al. The prevalence rate of anxiety and depressiveness was found to be higher in women, which probably reflects the gender difference in anxiety and depressive symptoms [34]. A study by Liu et al. found that nursing staff showed higher prevalence estimates for both anxiety and depressive symptoms compared with physicians. These findings may be partially challenged by the fact that nurses are predominantly female, but may also be attributed to the fact that they may be at higher risk of contracting infections from COVID-19 patients because they spend more time on the wards, provide direct patient care and are responsible for collecting sputum for viral detection [35]. Unfortunately, there have also been reports of suicide, as healthcare workers deal with accumulated psychological pressure and intense fear of death [36,37]. This is particularly worrisome given that healthcare personnel are already at an increased risk for suicide compared with the general population [38].

Initially, data from previous epidemics, most notably the SARS epidemic, were considered when organizing psychological support due to the lack of current research findings. Data on the negative consequences of isolation during SARS were also highlighted. The most common direct effects of the 9-day quarantine in medical services included experiencing emotions and conditions such as exhaustion, irritability, anxiety, withdrawal from relationships with others, insomnia, attention deficit disorder and impaired occupational functioning, including considering quitting work [39].

The results of our study showed that almost half of the subjects were in quarantine. People in quarantine showed higher levels of anxiety as measured by GAD-7 than those who did not have this experience. Significantly, the effects of quarantine were a predictor of symptoms of post-traumatic stress disorder within 3 years of the outbreak [40]. After the quarantine period, medical staff were reported to have persistent symptoms of avoidant, protective behaviors, such as avoiding crowded rooms, public places or contact with people with signs of infection [41]. The outreach organized for medical personnel during the COVID-19 pandemic was initially built on experiences and guidelines from the SARS outbreak. One recommendation was for screening for depression, anxiety and suicide risk for medical personnel, as for those infected or awaiting test results, especially since the experience of the SARS outbreak indicated significant stress among medical personnel, continuing even one year after the outbreak. Based on the experience of previous epidemics and extensive studies of various population groups, a mental health strategy was developed in China early in the COVID-19 epidemic [42,43].

Pappa et al. [44] conducted a systematic review and meta-analysis of the prevalence of anxiety, depression and insomnia among healthcare workers during the COVID-19 pandemic. Anxiety was assessed in 12 studies, with a prevalence of 23.2%; depression was assessed in 10 studies, with a prevalence of 22.8%. Labrague and De Los Santos [45] found that 37.8% of nurses surveyed had dysfunctional levels of anxiety. Labrague and De Los Santos indicated that COVID-19 anxiety was associated with social support, organizational support and personal resilience. These findings support the current study showing that frontline nurses were affected by anxiety during the COVID-19 pandemic. To help healthcare workers provide care in extremely challenging clinical settings, such as the COVID-19 pandemic, emotional and behavioral responses among workers should be recognized and enhanced through education and training to overcome fear and empathic distress [46].

A study by Alnazly et al. demonstrated the presence of fear, depression, anxiety and stress among healthcare workers during the COVID-19 pandemic. Workers surveyed identified social support from family and friends as important during the pandemic and demonstrated the need for increased social support to adjust to psychological stress. Factors found to be associated with psychological distress were male gender, aged 40 years or older and having a life partner or more clinical experience [47].

Published data strongly suggest that the health status of medical professionals is significantly impacted due to the ongoing COVID-19 pandemic. The clinical picture in this population shows an increase in depressive symptoms, anxiety and insomnia [41]. Psychological support, access to up-to-date data on new treatment options and adequate comfort by matching staff to patients has a positive impact on health care and will reduce errors that can have dramatic consequences [35]. It is essential to ensure the safety of workers through the availability of personal protective equipment, i.e., disinfectants, masks, goggles, visors, protective shoes and protective suits, that meet all required standards. These reports are very alarming. They indicate the need to build a strategy of mental health protection, undertake more intensive preventive actions toward medical workers and monitor the state of the mental health of this professional group after the end of the pandemic.

### Limitations

Although the literature on this matter is scarce, a few studies that analyzed the nurses’ psychological functioning (which assessed the frequency of depression, anxiety, insomnia, the level of perceived stress or the assessment of experienced traumatic events according to the Impact of Events Scale) have already been published during the COVID-19 pandemic. Unfortunately, our study had some limitations due to the extensive questionnaire administered to nurses. In Poland, there has been a shortage of nursing staff in hospitals for years, and due to the COVID-19 pandemic, nurses felt this shortage even more strongly; therefore, obtaining willing nurses to participate in the study was quite difficult. In addition, our study focused on employees of only one hospital in Szczecin, and it was a cross-sectional study, which means we cannot assess the long-term consequences of the COVID-19 pandemic on the mental health of healthcare workers. Despite the limitations of our study, it is worth mentioning that its advantage was the individual approach to the studied professional group. Therefore, it is advisable to conduct further research and take preventive measures to protect the mental health of nursing staff.

## 5. Conclusions

1. Education, gender and age were the variables that significantly affected the severity of anxiety, depression and insomnia of the studied nurses working with patients with COVID-19.
2. The form of employment was one of the factors that influenced the intensity of anxiety of the studied nurses.
3. Along with the intensity of intrusion in the studied nurses also increased the level of anxiety, depression and stress.
4. The positive self-assessment of the health of the surveyed nurses had a significant impact on the reduction of anxiety and depression during the implementation of care for patients with COVID-19.

## Figures and Tables

**Table 1:** Influence of sociodemographic variables on the occurrence of insomnia according to the AIS.

Factor		b	OR	−95% CI	+95% CI	t	*p*
Intercept		0.031	1.031	0.093	11.458	0.001	0.980
Gender	Female (ref.)						
	Male	−0.745	0.475	0.197	1.145	2.751	0.097
Age		−0.042	0.959	0.924	0.994	5.170	0.023
Marital status	Single (ref.)						
	Formal relationship	−0.409	0.664	0.287	1.541	0.908	0.341
	Casual relationship	−0.411	0.663	0.275	1.600	0.835	0.361
Place of residence	Rural area (ref.)						
	Small town	0.203	1.225	0.384	3.908	0.118	0.732
	Big city	−0.241	0.786	0.275	2.249	0.201	0.654
Education	Secondary/ post-secondary education (ref)						
	Higher	0.098	1.103	0.459	2.652	0.048	0.826
Number of children	None (ref.)						
	One	0.553	1.738	0.750	4.025	1.663	0.197
	Two and more	0.479	1.614	0.595	4.382	0.884	0.347
Type of employment	Employment contract	1.180	3.254	0.931	11.374	3.416	0.065
	Self-employment	0.675	1.964	0.527	7.323	1.011	0.315
	Contract of mandate	0.976	2.655	0.937	7.518	3.379	0.066
Shift work	No (ref.)						
	Yes	0.360	1.434	0.749	2.745	1.182	0.277

b—regression coefficient, OR—odds ratio, CI—confidence interval, ref.—reference level.

**Table 2:** The influence of the self-assessment of health, quarantine, psychological support, presence of chronic diseases and dimensions according to the Impact of Event Scale—Revised (IES-R) on the occurrence of insomnia according to the AIS.

Factor		b	OR	−95% CI	+95% CI	t	*p*
Intercept		−2.044	0.130	0.026	0.647	6.203	0.013
Quarantine	No (ref.)						
	Yes	0.266	1.305	0.570	2.985	0.396	0.529
Psychologist support	No (ref.)						
	Yes	0.261	1.298	0.662	2.545	0.577	0.448
Self-assessed health status	Poor/average (ref.)						
	Good	−0.607	0.545	0.154	1.934	0.882	0.348
	Very good	−0.755	0.470	0.115	1.918	1.108	0.293
Chronic disease	No (ref.)						
	Yes	−0.226	0.798	0.386	1.649	0.372	0.542
IES-R	Intrusion	0.066	1.068	0.985	1.157	2.553	0.110
	Stimulation	0.196	1.217	1.075	1.377	9.649	0.002
	Avoiding	−0.083	0.920	0.836	1.012	2.921	0.087

b—regression coefficient, OR—odds ratio, CI—confidence interval, ref.—reference level, IES-R—Impact of Event Scale—Revised.

**Table 3:** Effect of the self-assessment of health, quarantine, psychological support, presence of chronic diseases and dimensions of the Impact of Event Scale—Revised (IES-R) on the level of anxiety according to GAD-7 (model IV).

Factor		b	β_stand._	−95% CI	+95% CI	t	*p*
Intercept		2.856				3.681	0.000
Quarantine	No (ref.)						
	Yes	0.841	0.123	0.015	0.231	2.242	0.026
Psychologist support	No (ref.)						
	Yes	−0.124	−0.023	−0.130	0.085	−0.416	0.678
Self-assessed health status	Poor/average (ref.)						
	Good	−0.851	−0.100	−0.208	0.009	−1.805	0.073
	Very good	−1.288	−0.132	−0.247	−0.018	−2.285	0.023
Chronic disease	No (ref.)						
	Yes	0.067	0.012	−0.098	0.121	0.207	0.836
IES-R	Intrusion	0.236	0.340	0.137	0.543	3.304	0.001
	Stimulation	0.316	0.366	0.132	0.600	3.085	0.002
	Avoiding	−0.087	−0.102	−0.276	0.072	−1.155	0.249

b—regression coefficient, β_stand._—standardized regression coefficient, CI—confidence interval, ref.—reference level, IES-R—Impact of Event Scale—Revised.

**Table 4:** Effect of self-rated health, quarantine, psychological support, presence of chronic diseases and the Impact of Event Scale—Revised (IES-R) dimensions on the level of depression according to the PHQ-9 (model V).

Factor		b	β_stand._	−95% CI	+95% CI	t	*p*
Intercept		2.908				3.287	0.001
Quarantine	No (ref.)						
	Yes	0.703	0.090	−0.018	0.198	1.643	0.102
Psychologist support	No (ref.)						
	Yes	−0.004	−0.001	−0.108	0.107	−0.011	0.991
Self-assessed health status	Poor/average (ref.)						
	Good	−1.009	−0.103	−0.211	0.005	−1.876	0.062
	Very good	−1.642	−0.147	−0.261	−0.034	−2.555	0.011
Chronic disease	No (ref.)						
	Yes	−0.579	−0.087	−0.196	0.022	−1.568	0.119
IES-R	Intrusion	0.308	0.387	0.185	0.589	3.781	0.000
	Stimulation	0.344	0.347	0.115	0.580	2.944	0.004
	Avoiding	−0.150	−0.153	−0.326	0.020	−1.740	0.083

b—regression coefficient, β_stand._—standardized regression coefficient, CI—confidence interval, ref.—reference level, IES-R—Impact of Event Scale—Revised.

**Table 5:** Effect of the self-assessment of health, quarantine, psychological support, presence of chronic diseases and dimensions of the Impact of Event Scale—Revised (IES-R) on stress level (model VI).

Factor		b	β_stand._	−95% CI	+95% CI	t	*p*
Intercept		6.039				24.030	0.000
Quarantine	No (ref.)						
	Yes	0.076	0.039	−0.083	0.161	0.627	0.531
Psychologist support	No (ref.)						
	Yes	0.049	0.031	−0.090	0.153	0.510	0.610
Self-assessed health status	Poor/average (ref.)						
	Good	−0.368	−0.149	−0.272	−0.027	−2.408	0.017
	Very good	−0.062	−0.022	−0.151	0.107	−0.338	0.735
Chronic disease	No (ref.)						
	Yes	0.016	0.009	−0.114	0.133	0.151	0.880
IES-R	Intrusion	0.056	0.281	0.053	0.510	2.430	0.016
	Stimulation	0.051	0.206	−0.057	0.470	1.545	0.124
	Avoiding	0.008	0.033	−0.163	0.229	0.330	0.742

b—regression coefficient, β_stand._—standardized regression coefficient, CI—confidence interval, ref.—reference level, IES-R—Impact of Event Scale—Revised.

## Data Availability

Data sharing not applicable.

## Supplementary Materials

The following supporting information can be downloaded at: https://www.mdpi.com/article/10.3390/ijerph19031435/s1, Table S1. STROBE statement—Checklist of items that should be included in reports of case–control studies.; Table S2. Sociodemographic variables of respondents; Table S3. Outcome measures: insomnia according to the AIS, generalized anxiety according to GAD-7, Impact of Events Scale (IES-R), depression according to the PHQ-9 and stress according to the PSS-10 in the entire cohort; Table S4. Outcome measures: insomnia according to the AIS, generalized anxiety according to GAD-7, Impact of Events Scale (IES-R), depressiveness according to the PHQ-9 and stress according to the PSS-10 in the entire cohort by category; Table S5. The influence of sociodemographic variables on the level of anxiety; Table S6. Influence of sociodemographic variables on the level of depression according to the Patient Health Questionnaire-9 (PHQ-9) (model II); Table S7. The influence of sociodemographic variables on the level of stress according to The Perceived Stress Scale (PSS-10) (model III).
