# Supplementary material for: Assessment of Psychosocial Functioning of Polish Nurses during COVID-19 Pandemic
Source: Int J Environ Res Public Health. 2022 Jan 27;19(3):1435. doi: 10.3390/ijerph19031435 (PMC8835236; doi:10.3390/ijerph19031435)
Supplement: Supplementary file 1 [file ijerph-19-01435-s001.zip › ijerph-1545184-supplementary-table s2-7.pdf]

Supplementary Materials

Table S2. Sociodemographic variables of respondents

Variables (N=207)		n	%
Gender	Female	172	83.09
	Male	35	16.91
Marital status	formal relationship	109	52.66
	casal relationship	57	27.54
	single	41	19.81
Education	Secondary/post-secondary education	43	20.77
	higher	164	79.23
Place of residence	Rural area	21	10.14
	City less then 100 t.	48	23.19
	City more then >100 tys.	138	66.67
	No	103	49.76
	yes	104	50.24
Number of children	none	103	49.76
	one	48	23.19
	Two and more	56	27.05
Type of employment	employment contract	139	67.15
	self-employment	62	29.95

	contract of mandate	29	14.01
Shift work	No	142	68.60
	Yes	65	31.40
N, whole cohort size, n, number of, %, percent			

Table S3. Outcome measures: insomnia by AIS, generalized anxiety by GAD-7, Impact of Events Scale (IES-R), depression by PHQ-9, and stress by PSS-10 in the entire cohort

Importance category (PKT)						
**Variables**	**M**	**SD**	**Me**	**IQR/2**	**Min-Max**	**CV [%]**
**AIS (sum of pts)**	6.71	4.39	6.00	3.50	0-22	65.41
**GAD-7 (sum of pts)**	7.12	5.41	6.00	4.00	0-21	76.03
**IES-R (sum of pts)**	34.25	19.65	36.00	15.50	0-88	57.38
**IES-R, i**ntrusion	12.34	7.78	12.00	6.00	0-32	63.05
**IES-R,** stimulating	11.58	6.26	11.00	4.50	0-28	54.05
**IES-R,** avoiding	12.12	6.31	12.00	4.50	0-28	52.10
**PHQ-9 (sum of pts)**	7.77	6.20	7.00	4.00	0-27	79.78
**PSS-10** (sten)	7.14	1.56	7.00	1.00	1-10	21.80
AIS, Athens Insomnia Scale, GAD-7, generalized anxiety disorder; IES-R, Impact of Event Scale - Revised ; PHQ-9, Patient Health Questionnaire-9; PSS-10, The Perceived Stress Scale; M- average Me- median SD - standard deviation IQR/2- quadrant gap; CV- Coefficient of variation						

Table S4. Outcome measures: insomnia by AIS, generalized anxiety by GAD-7, Impact of Events Scale (IES-R), depressiveness by PHQ-9, and stress by PSS-10 in the entire cohort by category

Importance category			
Variables (pts)		n	%
AIS	absence of insomnia	123	59.42
	insomnia	84	40.58
GAD-7	absence of anxiety	71	34.30
	mild anxiety	76	36.71
	moderate anxiety	41	19.81
	strong anxiety	19	9.18
PSS-10	small (1-4 steny)	13	6.28
	moderate (5-6 stena)	43	20.77
	high (7-10 stena)	151	72.95
PHQ-9	absence of depression	77	37.20
	moderate of depression	64	30.92
	depression	66	31.88

n, number of, %, procent, AIS, Athens Insomnia Scale, GAD-7, generalized anxiety disorder; PSS-10, The Perceived Stress Scale; PHQ-9, Patient Health Questionnaire-9;

Table S5. The influence of sociodemographic variables on the level of anxiety according to Generalized Anxiety Disorder (GAD-7) (model I).

**Factor**		**b**	**$\beta_{stand.}$**	**-95% CI**	**+95% CI**	**t**	**p**
Intercept		9.715				5.122	0.000
Gender	Female (ref.)						
	Male	-0.752	-0.105	-0.241	0.032	-1.512	0.132
Age		-0.062	-0.137	-0.319	0.045	-1.479	0.141
Marital status	Single (ref.)						
	Formal relationship	-0.018	-0.003	-0.161	0.156	-0.033	0.974
	Casual relationship	0.129	0.016	-0.130	0.162	0.221	0.825
Place of residence	Rural area (ref.)						
	Small town	0.858	0.089	-0.047	0.226	1.289	0.199
	Big city	-0.887	-0.110	-0.245	0.025	-1.607	0.110
Education	Secondary/ Post-secondary education (ref.)						
	Higher	**1.267**	**0.190**	**0.038**	**0.342**	**2.472**	**0.014**
Number of children	None (ref.)						
	One	-0.225	-0.034	-0.208	0.140	-0.384	0.702
	Two and more	0.674	0.106	-0.103	0.314	0.999	0.319
Type of employment	Employment contract	**2.299**	**0.400**	**0.143**	**0.658**	**3.065**	**0.002**
	Self-employment	**1.929**	**0.328**	**0.064**	**0.591**	**2.448**	**0.015**
	Contract of mandate	**1.318**	**0.169**	**0.011**	**0.328**	**2.106**	**0.037**
Shift work	No (ref.)						
	Yes	-0.270	-0.047	-0.182	0.087	-0.693	0.489

b -regression coefficient, $\beta_{stand.}$ - standardized regression coefficient, CI – confidence interval, ref. – reference level

Table S6. Influence of sociodemographic variables on the level of depression according to Patient Health Questionnaire-9 (PHQ-9) (model II).

Factor		b	$\beta_{stand.}$	-95% CI	+95% CI	t	p
Intercept		9.889				4.485	0.000
Gender	Female (ref.)						
	Male	**-1.267**	**-0.154**	**-0.292**	**-0.015**	**-2.190**	**0.030**
Age		-0.046	-0.089	-0.273	0.096	-0.946	0.345
Marital status	Single (ref.)						
	Formal relationship	-0.582	-0.074	-0.235	0.087	-0.906	0.366
	Casual relationship	0.270	0.030	-0.118	0.178	0.398	0.691
Place of residence	Rural area (ref.)						
	Small town	0.207	0.019	-0.120	0.158	0.267	0.790
	Big city	-0.401	-0.043	-0.181	0.094	-0.625	0.532
Education	Secondary/post-secondary education (ref.)						
	Higher	0.801	0.105	-0.049	0.259	1.345	0.180
Number of children	None (ref.)						
	One	-0.119	-0.016	-0.192	0.161	-0.174	0.862
	Two and more	0.445	0.061	-0.151	0.273	0.568	0.571
Type of employment	Employment contract	**2.734**	**0.415**	**0.154**	**0.677**	**3.136**	**0.002**
	Self-employment	**2.017**	**0.299**	**0.031**	**0.567**	**2.202**	**0.029**
	Contract of mandate	**1.942**	**0.218**	**0.057**	**0.379**	**2.670**	**0.008**
Shift work	No (ref.)						
	Yes	0.050	0.008	-0.129	0.144	0.111	0.912
b – regression coefficient, $\beta_{stand.}$ – standardized regression coefficient, CI – confidence interval, ref. – reference level							

Table S7. The influence of sociodemographic variables on the level of stress according to The Perceived Stress Scale (PSS-10) (model III).

Factor		b	$\beta_{stand.}$	-95% CI	+95% CI	t	p
Intercept		7.158				12.621	0.000
Gender	Female (ref.)						
	Male	-0.037	-0.018	-0.160	0.124	-0.250	0.803
Age		-0.002	-0.016	-0.205	0.174	-0.163	0.871
Marital status	Single (ref.)						
	Formal relationship	-0.158	-0.080	-0.245	0.085	-0.957	0.340
	Casual relationship	0.143	0.063	-0.088	0.215	0.821	0.412
Place of residence	Rural area (ref.)						
	Small town	-0.099	-0.036	-0.178	0.106	-0.497	0.620
	Big city	0.270	0.116	-0.024	0.257	1.634	0.104
Education	Secondary/post-secondary education (ref.)						
	Higher	0.210	0.110	-0.048	0.268	1.372	0.172
Number of children	None (ref.)						
	One	-0.055	-0.029	-0.210	0.152	-0.312	0.755
	Two and more	0.172	0.094	-0.123	0.310	0.850	0.396
Type of employment	Employment contract	**0.573**	**0.347**	**0.079**	**0.615**	**2.557**	**0.011**
	Self-employment	0.379	0.223	-0.051	0.498	1.606	0.110
	Contract of mandate	0.355	0.159	-0.006	0.324	1.897	0.059
Shift work	No (ref.)						
	Yes	0.142	0.086	-0.054	0.226	1.217	0.225

b – regression coefficient, $\beta_{stand.}$ – standardized regression coefficient, CI – confidence interval, ref. – reference level
